# Polypill for atherosclerotic cardiovascular disease prevention in Haiti: Eligibility estimates in a low-income country

**DOI:** 10.3389/fepid.2022.925464

**Published:** 2022-07-14

**Authors:** Lily D. Yan, Vanessa Rouzier, Jean Lookens Pierre, Eliezer Dade, Rodney Sufra, Mark D. Huffman, Alexandra Apollon, Stephano St Preux, Miranda Metz, Shalom Sabwa, Béatrice Morisset, Marie Deschamps, Jean W. Pape, Margaret L. McNairy

**Affiliations:** 1 Division of General Internal Medicine, Department of Medicine, Weill Cornell Medicine, New York, NY, United States; 2 Center for Global Health, Weill Cornell Medicine, New York, NY, United States; 3 Haitian Group for the Study of Kaposi’s Sarcoma and Opportunistic Infections (GHESKIO), Port-au-Prince, Haiti; 4 Cardiovascular Division and Global Health Center, Department of Medicine, Washington University in St Louis, St Louis, MO, United States; 5 The George Institute for Global Health, University of New South Wales, Sydney, NSW, Australia; 6 Collège Haïtien de Cardiologie, Port-au-Prince, Haiti

**Keywords:** Haiti, low-middle income country, global health, preventive cardiology, epidemiology

## Abstract

**Background:**

Multidrug therapy is a World Health Organization “best buy” for the prevention and control of noncommunicable diseases. CVD polypills, including ≥2 blood pressure medications, and a statin with or without aspirin, are an effective, scalable strategy to close the treatment gap that exists in many low- and middle-income countries, including Haiti. We estimated the number of Haitian adults eligible for an atherosclerotic CVD (ASCVD) polypill, and the number of potentially preventable CVD events if polypills were implemented nationally.

**Methods:**

We used cross-sectional data from the Haiti CVD Cohort, a population-based cohort of 3,005 adults ≥18 years in Port-au-Prince, to compare two polypill implementation strategies: high-risk primary prevention and secondary prevention. High-risk primary prevention included three scenarios: (a) age ≥40 years, (b) hypertension, or (c) predicted 10-year ASCVD risk ≥7.5%. Secondary prevention eligibility included history of stroke or myocardial infarction. We then used the 2019 Global Burden of Disease database and published polypill trials to estimate preventable CVD events, defined as nonfatal MI, nonfatal stroke, and cardiovascular death over a 5-year timeline.

**Results:**

Among 2,880 participants, the proportion of eligible adults for primary prevention were: 51.6% for age, 32.5% for hypertension, 19.3% for high ASCVD risk, and 5.8% for secondary prevention. Based on current trends, an estimated 462,509 CVD events (95% CI: 369,089–578,475) would occur among adults ≥40 years in Haiti from 2019–2024. Compared with no polypill therapy, we found 32% or 148,003 CVD events (95% CI: 70,126–248,744) could be prevented by a combined primary and secondary prevention approach in Haiti if polypills were fully implemented over 5 years.

**Conclusion:**

These modeling estimates underscore the potential magnitude of preventable CVD events in low-income settings like Haiti. Model calibration using observed CVD events, costs, and implementation assumptions are future directions.

**Clinical trial registration:**

clinicaltrials.gov, identifier: NCT03892265.

## Introduction

Cardiovascular diseases (CVD) are the leading cause of morbidity and mortality in low- and middle-income countries (LMICs), with 4.6 million deaths in 2019, yet large gaps in modifiable risk factor control for hypertension and hyperlipidemia persist (1–3). Atherosclerotic CVD polypills, which are fixed-dose combinations of low doses of two or more blood pressure (BP) medications and a statin with or without aspirin, have been shown to be effective at reducing CVD risk (4). In randomized trials across high and low-income settings and populations, ASCVD polypills lower BP, low density lipoprotein cholesterol (LDLc), and CVD events (5–9).

Patients who may benefit from ASCVD polypills are typically categorized into high-risk primary or secondary CVD prevention (10). Primary prevention strategies identify individuals more likely to benefit based on a single risk factor (e.g., age, raised BP) or based on predicted, multivariable CVD risk scores (10). Secondary prevention targets a narrower population among individuals with history of ASCVD events, like stroke or myocardial infarction (MI).

We estimated the potential impact of ASCVD polypills in Haiti, a low-income country where CVD is the leading cause of adult mortality (11) and hypertension prevalence is 29%, but only 13% have controlled BP (12). We explored the number of adults who would be eligible for polypills based on high-risk primary prevention and secondary prevention. We then estimated the potential number of preventable CVD events nationally with each strategy.

## Materials and methods

### Study design and data sources

Our study design combines a cross-sectional analysis with a modeling estimation. We compared two ASCVD polypill implementation strategies: high-risk primary and secondary prevention (10). Primary prevention included three eligibility scenarios: (a) age ≥40 years, (b) essential hypertension defined as BP ≥140/90 mm Hg per World Health Organization (WHO) guidelines, and (c) predicted 10-year CVD high risk ≥7.5% calculated by the Pooled Cohort Equations (13). Although other polypill trials used older age thresholds for primary prevention (7), we chose age ≥40 given the Haitian population is very young [48% is <40 years, life expectancy = 64 years (14)], and ASCVD risk prediction begins at age 40. Secondary prevention included eligibility based on history of prior stroke or MI. Polypill trial data are listed in Supplementary Table S1.

The primary outcome of this analysis was the proportion of Haitian adults eligible for a ASCVD polypill under various scenarios. The secondary outcome was the number of potentially preventable CVD events under national implementation of various prevention strategies over a 5-year timeline in Haiti assuming static population size, population structure, risk factor levels, and background event rate.

For the primary outcome, we used cross-sectional enrollment data within the Haiti CVD Cohort Study, a longitudinal population-based cohort of 3,005 adults living in Port-au-Prince that aims to measure prevalence and incidence of CVD and risk factors (15). This cohort is one of the first population-based studies in Haiti that systematically measured laboratory values including serum lipids. We included all participants ≥18 years, with enrollment from March 2019 to August 2021. Participants missing required data for ASCVD risk estimation were excluded (*n* = 125, 4.2%).

For the secondary outcome, we used the 2019 Global Burden of Disease (GBD) estimates of incident CVD events in Haiti. CVD events included nonfatal MI, nonfatal stroke, and CVD death (16). We then used results from published randomized controlled trials of ASCVD polypills to determine the hazard ratio for CVD events in adults receiving the polypill vs. placebo. Given lack of dedicated trial data for secondary prevention, we used the same hazard ratio for a combined primary and secondary prevention approach from an individual participant meta-analysis on polypill use for CVD prevention (polypill without aspirin, HR 0.68, 95% CI 0.57–0.81), including one study using treatment based on age with additional clinical criteria regardless of prior CVD events, and two using treatment based on high predicted 10-year ASCVD risk (9).

### Measurements

We collected sociodemographic data, medical history, health behaviors, and a clinical exam during an enrollment survey using standardized World Health Organization (WHO) STEPs instruments (17). Three unobserved BP measurements were taken according to WHO guidelines with an Omron HEM-905, with the second and third BP measurements averaged for all analyses (17).

Hypertension was defined as any of the following: systolic BP (SBP) ≥140 mmHg, diastolic BP (DBP) ≥90 mmHg, on hypertension medications, or clinician diagnosis of hypertension based on WHO thresholds (17). Hypercholesterolemia was defined as any of the following: low density lipoprotein cholesterol ≥160 mg/dl, on a statin, or clinician diagnosis of hypercholesterolemia (18). Diabetes mellitus was defined as any of the following: random glucose ≥200 mg/dl, fasting glucose ≥126 mg/dl, on diabetes medications, or clinician diagnosis (19). CVD history was measured as self-reported history of stroke or MI.

### Statistical analysis

We summarized the proportion of the Haiti CVD Cohort that was polypill eligible under primary prevention and secondary prevention scenarios with descriptive statistics. We followed four steps to estimate potentially preventable ASCVD events. First, we assumed a timeline of 5 years given polypill trial data have a median follow up period of 5 years (9). Second, we used 2019 GBD estimates of incident CVD events for adults ≥40 years to calculate the number of incident CVD events over 5 years, assuming static population size and structure and without any changes in risk factor levels or annual rates of incident events. Third, we calculated incident and recurrent CVD events under nationwide polypill implementation under a combined primary and secondary prevention approach by applying clinical trial hazard ratios and corresponding 95% confidence intervals to GBD estimates of 5-year incident CVD events. Lastly, we calculated preventable CVD events by subtracting the number of incident and recurrent CVD events under a national polypill strategy from the estimated number of CVD events without polypill implementation.

### Ethics

This study was approved by institutional review boards at Weill Cornell Medicine and Groupe Haitien d’Etude du Sarcome de Kaposi et des Infections Opportunistes (GHESKIO) (1803019037), with written participant consent.

## Results

### Characteristics of polypill target groups under implementation strategies

Out of 3,005 participants, 2,880 (95.8%) had complete data and were included (Supplementary Figure S1). Table 1 describes the participants’ demographics and proportion of the cohort who would be polypill eligible under various primary or secondary prevention strategies. Sex-stratified demographics are reported in Supplementary Table S2.

### Proportion of Haiti CVD cohort eligible for a ASCVD polypill

**FIGURE 1:**
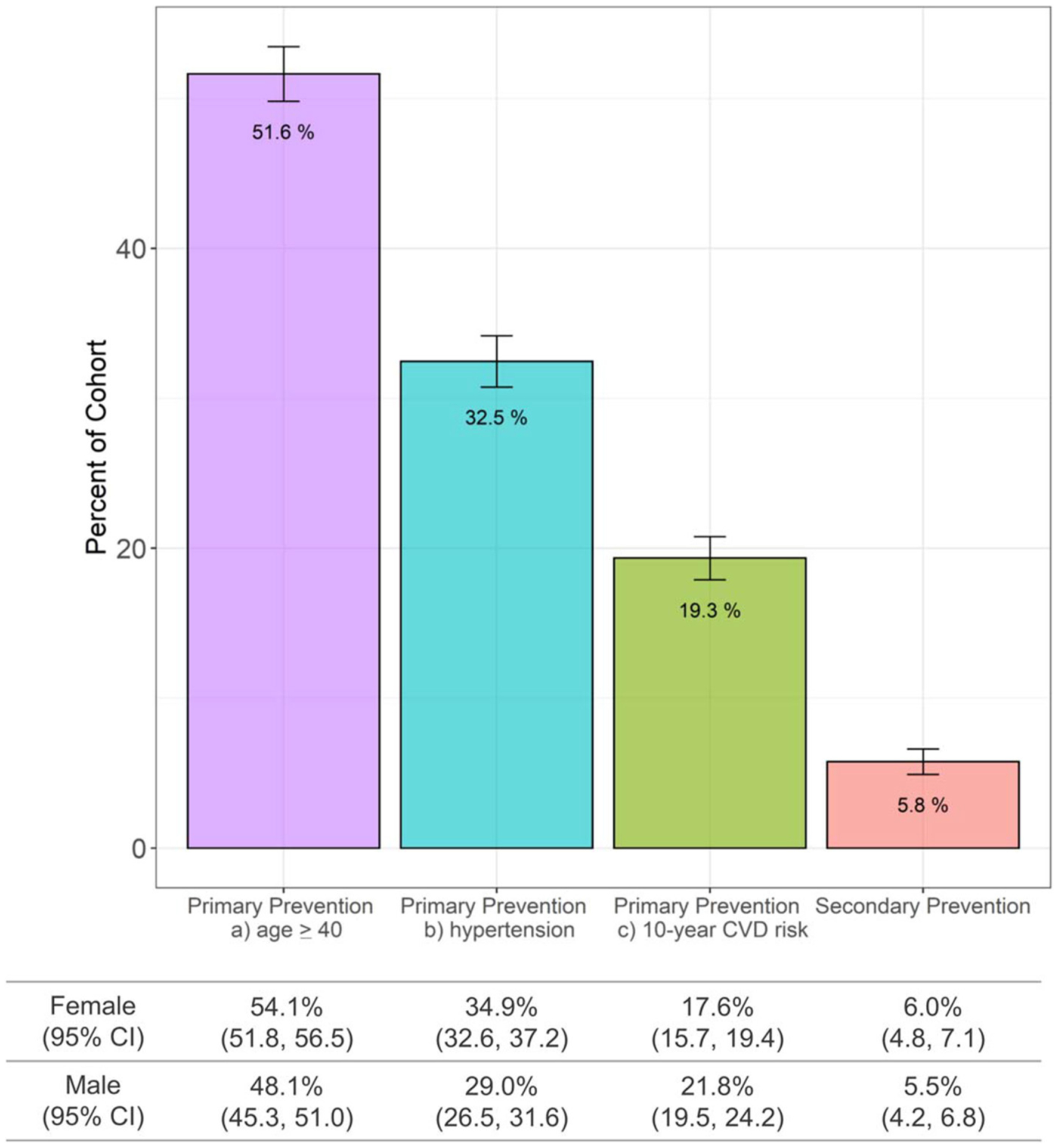
ASCVD polypill eligibility in Haiti using the Haiti CVD Cohort (*N* = 2,880 participants age ≥18 years). ASCVD, atherosclerotic cardiovascular disease; CI, confidence intervals; CVD, cardiovascular disease.

The highest proportion (51.6%, 1,487/2,880) of adults in the Haiti CVD Cohort were eligible for an ASCVD polypill under a primary prevention strategy using eligibility scenario of age ≥40 years (Figure 1). Under primary prevention based on hypertension, 32.5% were eligible, while primary prevention based on high 10-year predicted ASCVD risk resulted in 19.3% being eligible. Using a secondary prevention strategy, 5.8% of adults within the Haiti CVD Cohort had a polypill indication, including 3.4% based on past MI and 2.6% on past stroke.

### Potentially preventable CVD events under nation-wide polypill strategy

Based on GBD estimates, over a 5-year period, 462,509 incident and recurrent CVD events (95% CI: 369,089–578,475) among people aged ≥40 years are predicted to occur assuming no changes in population size, structure, disease incidence, or risk factor levels (Table 2). Using the hazard ratio from published ASCVD polypill trials, 148,003 CVD events (95% CI: 70,126–248,744) or 32% could be potentially preventable with a nation-wide polypill strategy based on a combined primary and secondary prevention strategy.

## Discussion

We estimate the potential impact of polypills for ASCVD prevention in Haiti, finding it could result in a 32% (95% CI: 15%−54%) reduction in CVD-related events that would have a substantial effect on the CVD epidemic in Haiti.

Premature CVD deaths continue to grow in LMIC, and epidemiological studies have found that modifiable risk factors such as hypertension, dyslipidemia, tobacco use, diet, physical inactivity, obesity, diabetes, psychological stress, and unhealthy alcohol use account for as much as 90% of CVD events (20, 21). ASCVD polypills are recommended by the WHO as a “best buy” for noncommunicable disease prevention and control (22).Polypills represent a pragmatic intervention and may be particularly relevant for fragile health systems where high costs of accessing repeated clinic-based care make medication titration challenging. Haiti is the poorest country in the Western Hemisphere, and CVD risk in Haiti is driven primarily by hypertension (23). In middle and high-income countries, polypills reduced systolic BP by 7–19 mm Hg, and LDLc by 11–14 mg/dl (5, 24, 25). These gains in CVD risk factor control have translated into longer-term reductions, including reduced CVD mortality, in varied settings from Canada to Tanzania (7, 9, 26).

This study uses some of the first available population-based CVD epidemiological data on prevalence of CVD risk factors in a low-income country to estimate the potential impact of ASCVD polypill implementation. Limitations include potentially over-optimistic hazard ratios based on randomized trials compared with general populations due to limited access to medical care, limited medication supplies or diagnostic capacity, and suboptimal adherence, and potential over-estimation of eligibility with age ≥40 years. Additional calibrations are needed to fine tune these estimates using adjudicated CVD events in Haiti and accounting for longitudinal changes in population size, structure, risk factors, and event rates. Implementation studies are needed to evaluate acceptability and feasibility of implementation and cost in Haiti, among other settings.

In summary, practical and effective interventions to reduce CVD risk are urgently needed in LMICs, which disproportionately bear the burden of CVD mortality. In this case study of a LMIC with high CVD mortality, a significant proportion of adults would be eligible for primary and secondary prevention with potentially large reductions in CVD events over time if implemented on a national level.

## Supplementary Material

Supplementary Material

## Figures and Tables

**TABLE 1:** Characteristics of adults eligible for ASCVD polypill in the Haiti CVD cohort.

	Primary prevention			Secondary prevention
	Scenario A Age ≥40 years	Scenario B Hypertension	Scenario C High 10-year CVD risk	
*N* (%)				
Total	1,487 (51.6%)	935 (32.5%)	557 (19.3%)	166 (5.8%)
Female	907 (61.0)	585 (62.6)	294 (52.8)	100 (60.2)
Age, median [IQR], year	54 [47, 62]	56 [47, 63]	62 [57, 68]	58 [44, 63]
Race, Black	1,487 (100%)	935 (100%)	557 (100%)	166 (100%)
Education, primary or lower	902 (60.7)	595 (63.6)	427 (76.7)	91 (54.8)
Income (daily), ≤1 USD	1,009 (67.9)	651 (69.6)	400 (71.8)	128 (77.1)
Smoking, current	57 (3.8)	33 (3.5)	39 (7.0)	4 (2.4)
Physical activity, ≤150 min/week (low)	848 (57.2)	543 (58.3)	332 (59.9)	81 (48.8)
Alcohol intake, more than 1 drink a day (moderate-high)	33 (2.2)	20 (2.1)	8 (1.4)	6 (3.6)
BMI, ≥30 kg/m^2^	332 (22.3)	232 (24.8)	94 (16.9)	41 (24.7)
BMI, median kg/m^2^	25.5 [22.1, 29.4]	26.0 [22.6, 29.9]	24.9 [21.9, 28.6]	25.4 [22.2, 29.7]
Cholesterol				
HDL cholesterol <40 mg/dl	318 (21.4)	180 (19.3)	128 (23.0)	39 (23.5)
LDL cholesterol ≥130 mg/dl	510 (34.3)	358 (38.3)	237 (42.5)	61 (36.7)
HDL median, mg/dl	48 [41, 57]	49 [42, 57]	47 [40, 55]	47 [40, 55]
LDL median, mg/dl	114 [91, 142]	118 [93, 145]	124 [98, 148]	113 [86, 149]
Blood pressure				
SBP ≥140 mm Hg	619 (41.6)	674 (72.1)	391 (70.2)	87 (52.4)
DBP ≥90 mm Hg	398 (26.8)	467 (49.9)	216 (38.8)	61 (36.7)
SBP median, mm Hg	133 [117, 151]	148 [138, 162]	150 [136, 165]	143 [123, 161]
DBP median, mm Hg	79 [70, 90]	89 [81, 97]	86 [77, 96]	85 [71, 95]
Comorbidities				
Hypertension	831 (55.9)	935 (100.0)	460 (82.6)	122 (73.5)
On treatment	322 (21.7)	340 (36.4)	200 (35.9)	63 (38.0)
Hypercholesterolemia	255 (17.1)	185 (19.8)	111 (19.9)	43 (25.9)
On treatment	21 (1.4)	19 (2.0)	0 (0)	7 (4.2)
Diabetes mellitus	142 (9.5)	110 (11.8)	87 (15.6)	20 (12.0)
On treatment	71 (4.8)	52 (5.6)	42 (7.5)	8 (4.8)

ASCVD, atherosclerotic cardiovascular disease; BMI, body mass index; CVD, cardiovascular disease; DBP, diastolic blood pressure; IQR, interquartile range, 25th to 75th percentile; SBP, systolic blood pressure.

**TABLE 2:** Potentially preventable CVD events over 5-year timeline under nation-wide polypill strategy in Haiti.

	Combined primary and secondary prevention strategy		
	Total (95% CI)	Female (95% CI)	Male (95% CI)
Hazard ratio of CVD events of polypill vs. placebo based on prior studies (9)	0.68 (0.57–0.81)	0.68 (0.57–0.81)	0.68 (0.57–0.81)
Estimated CVD events in Haiti over 5 year timeline in age ≥40 years (death from CVD, incident nonfatal MI, incident nonfatal stroke) (9)	462,509 (369,089–578,475)	250,046 (196,042–316,387)	212,463 (166,385–271,408)
Potentially preventable CVD events with nation-wide polypill strategy	148,003 (70,126–248,744)	80,015 (37,248–136,046)	67,988 (31,613–116,705)

CI, confidence intervals; CVD, cardiovascular disease.

## Data Availability

Deidentified data used for this analysis are available upon request after signing a data access and use agreement, provision of approval by the GHESKIO ethics board, and demonstration that the external investigative team is qualified and has documented evidence of human research protection training. Requests to access the datasets should be directed at: LY, liy9032@med.cornell.edu.
