# Supplementary material for: Polypill for atherosclerotic cardiovascular disease prevention in Haiti: Eligibility estimates in a low-income country
Source: Front Epidemiol. 2022 Jul 14;2:925464. doi: 10.3389/fepid.2022.925464 (PMC9937442; doi:10.3389/fepid.2022.925464)
Supplement: Supplementary file 1 [file Data_Sheet_1.docx]

Supplementary Material

Polypill for atherosclerotic cardiovascular disease prevention in Haiti: eligibility estimates in a low-income country

**Lily D Yan^1,2^, Vanessa Rouzier^2,3^, Jean Lookens Pierre^3^, Eliezer Dade^3^, Rodney Sufra^3^, Mark D Huffman^4,5^, Alexander Apollon^3^, Stephano St Preux^3^, Miranda Metz^2^, Shalom Sabwa^2^, Béatrice Morisset^6^, Marie Deschamps^3^, Jean W Pape^3^, Margaret L McNairy^1,2^**

1. Division of General Internal Medicine, Department of Medicine, Weill Cornell Medicine, New York, New York, USA

2. Center for Global Health, Weill Cornell Medicine, New York, New York, USA

3. Haitian Group for the Study of Kaposi's Sarcoma and Opportunistic Infections (GHESKIO), Port-au-Prince, Haiti

4. Cardiovascular Division and Global Health Center, Department of Medicine, Washington University in St Louis, St Louis, Missouri, USA

5. The George Institute for Global Health, University of New South Wales, Sydney, Australia

6. Collège Haïtien de Cardiologie, Port-au-Prince, Haiti

*** Correspondence:**Lily D Yan
liy9032@med.cornell.edu

Keywords: Haiti, low-middle income country, global health, preventive cardiology, epidemiology

**Supplemental Figure 1: Flowchart of participants and data.**


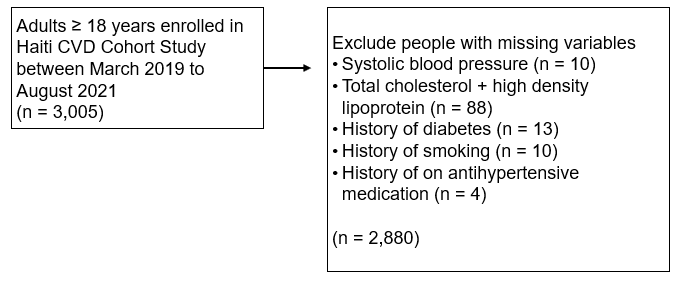


**Supplemental Table 1: Published clinical trials on ASCVD polypill implementation strategies.**

| **Implementation Strategy** | **Published trials** |
| --- | --- |
| Primary prevention based on simplified risk factors (age and clinical criteria) | PolyIran (1), Muñoz (2), Wald (3) |
| Primary prevention based on high predicted risk of CVD using formal calculation | TIPS-3 (4), HOPE-3 (5), CUSP (6), Malekzadeh (7), OLSTA (8), PILL Pilot (9), Soliman (10), TIPS (11), TOGETHER (12) |
| Secondary prevention among people with history of CVD | CRUCIAL (13), UMPIRE (14), IMPACT (15), Kanyini GAP (16), TIPS-2(17), FOCUS (18) |

**Supplemental Table 2: Characteristics of adults eligible for ASCVD polypill in the Haiti CVD Cohort, stratified by sex.**

|  | **Primary Prevention** | | | | | | **Secondary Prevention** | |
| --- | --- | --- | --- | --- | --- | --- | --- | --- |
|  | Scenario A  Age ≥40 years | | Scenario B  Hypertension | | Scenario C  High 10-year CVD Risk | |  |  |
|  | Females | Males | Females | Males | Females | Males | Females | Males |
|  | N (%) | | | | | | | |
| Total | 907 | 580 | 585 | 350 | 294 | 263 | 100 | 66 |
| **Age**, median [IQR], y | 54  [47, 62] | 55  [48, 63] | 55  [47, 62] | 58  [48, 65] | 62  [57, 68] | 62  [57, 68] | 56  [45, 63] | 59  [43, 63] |
| **Education,** primary or lower | 617 (68.0) | 285 (49.1) | 407 (69.6) | 188 (53.7) | 246 (83.7) | 181 (68.8) | 61 (61.0) | 30 (45.5) |
| **Income (daily),** ≤1 USD | 612 (67.5) | 397 (68.4) | 396 (67.7) | 255 (72.9) | 206 (70.1) | 194 (73.8) | 78 (78.0) | 50 (75.8) |
| **Smoking,** current | 31 (3.4) | 26 (4.5) | 23 (3.9) | 10 (2.9) | 20 (6.8) | 19 (7.2) | 2 (2.0) | 2 (3.0) |
| **Physical activity,** ≤ 150 min / week (low) | 512 (56.6) | 336 (58.1) | 328 (56.3) | 215 (61.8) | 169 (57.7) | 163 (62.5) | 46 (46.0) | 35 (53.0) |
| **Alcohol intake,** more than 1 drink a day (moderate-high) | 2 (0.2) | 31 (5.4) | 5 (0.9) | 15 (4.3) | 1 (0.3) | 7 (2.7) | 1 (1.0) | 5 (7.6) |
| **BMI,** ≥30 kg/m2 | 295 (32.5) | 37 (6.4) | 207 (35.4) | 25 (7.1) | 80 (27.2) | 14 (5.3) | 37 (37.0) | 4 (6.1) |
| BMI, median kg/m^2^ | 27.2 [23.3, 31.5] | 23.3 [21.0, 26.1] | 27.9 [24.0, 31.8] | 23.6 [21.4, 26.5] | 26.5 [23.2, 30.6] | 23.4 [21.2, 26.1] | 27.4 [23.3, 31.4] | 23.1 [20.6, 25.9] |
| **Cholesterol** |  |  |  |  |  |  |  |  |
| HDL cholesterol < 40 mg/dL | 157 (17.3) | 161 (27.8) | 87 (14.9) | 93 (26.6) | 51 (17.3) | 77 (29.3) | 25 (25.0) | 14 (21.2) |
| LDL cholesterol ≥130 mg/dL | 372 (41.0) | 138 (23.8) | 265 (45.3) | 93 (26.6) | 160 (54.4) | 77 (29.3) | 40 (40.0) | 21 (31.8) |
| HDL median, mg/dL | 49 [43, 58] | 46 [38, 55] | 49 [43, 58] | 47 [38, 55] | 48 [42, 56] | 46 [37, 55] | 46 [40, 55] | 48 [41, 55] |
| LDL median, mg/dL | 122 [98, 148] | 103 [83, 128] | 126 [99, 149] | 105 [85, 134] | 133 [112, 156] | 107 [87, 134] | 114 [89, 152] | 107 [81, 142] |
| **Blood Pressure** |  |  |  |  |  |  |  |  |
| SBP ≥140 mmHg | 376 (1.5) | 243 (41.9) | 405 (69.2) | 269 (76.9) | 220 (74.8) | 171 (65.0) | 54 (54.0) | 33 (50.0) |
| DBP ≥90 mmHg | 246 (27.1) | 152 (26.2) | 292 (49.9) | 175 (50.0) | 113 (38.4) | 103 (39.2) | 41 (41.0) | 20 (30.3) |
| SBP median, mm Hg | 133 [115, 151] | 134 [118, 152] | 147 [135, 160] | 149 [141, 166] | 152 [140, 169] | 147 [133, 164] | 143 [121, 162] | 140 [124, 156] |
| DBP median, mm Hg | 79 [71, 90] | 79 [69, 91] | 89 [80, 97] | 90 [82, 97] | 85 [77, 95] | 86 [77, 96] | 86 [72, 97] | 81 [71, 93] |
| **Comorbidities** |  |  |  |  |  |  |  |  |
| Hypertension | 519 (7.2) | 312 (53.8) | 585 (100.0) | 350 (100.0) | 254 (86.4) | 206 (78.3) | 71 (71.0) | 51 (77.3) |
| On treatment | 206 (2.7) | 116 (20.0) | 222 (37.9) | 118 (33.7) | 116 (39.5) | 84 (31.9) | 38 (38.0) | 25 (37.9) |
| Hypercholesterolemia | 180 (9.8) | 75 (12.9) | 135 (23.1) | 50 (14.3) | 77 (26.2) | 34 (12.9) | 28 (28.0) | 15 (22.7) |
| On treatment | 13 (1.4) | 8 (1.4) | 12 (2.1) | 7 (2.0) | 0 (0) | 0 (0) | 4 (4.0) | 3 (4.5) |
| Diabetes mellitus | 92 (10.1) | 50 (8.6) | 72 (12.3) | 38 (10.9) | 50 (17.0) | 37 (14.1) | 16 (16.0) | 4 (6.1) |
| On treatment | 44 (4.9) | 27 (4.7) | 32 (5.5) | 20 (5.7) | 23 (7.8) | 19 (7.2) | 6 (6.0) | 2 (3.0) |

Legend: ASCVD=atherosclerotic cardiovascular disease; BMI=body mass index; CVD=cardiovascular disease; DBP=diastolic blood pressure; IQR = interquartile range, 25^th^ to 75^th^ percentile; SBP=systolic blood pressure.

**References**

1. Roshandel G, Khoshnia M, Poustchi H, Hemming K, Kamangar F, Gharavi A, et al. Effectiveness of polypill for primary and secondary prevention of cardiovascular diseases (PolyIran): a pragmatic, cluster-randomised trial. The Lancet. 2019 Aug 24;394(10199):672–83.

2. Muñoz D, Uzoije P, Reynolds C, Miller R, Walkley D, Pappalardo S, et al. Polypill for Cardiovascular Disease Prevention in an Underserved Population. N Engl J Med. 2019 19;381(12):1114–23.

3. Wald DS, Morris JK, Wald NJ. Randomized Polypill Crossover Trial in People Aged 50 and Over. PLOS ONE. 2012 Jul 18;7(7):e41297.

4. Yusuf S, Joseph P, Dans A, Gao P, Teo K, Xavier D, et al. Polypill with or without Aspirin in Persons without Cardiovascular Disease. New England Journal of Medicine [Internet]. 2020 Nov 13 [cited 2021 Oct 12]; Available from: https://www.nejm.org/doi/10.1056/NEJMoa2028220

5. Yusuf S, Lonn E, Pais P, Bosch J, López-Jaramillo P, Zhu J, et al. Blood-Pressure and Cholesterol Lowering in Persons without Cardiovascular Disease. New England Journal of Medicine. 2016 May 26;374(21):2032–43.

6. Neutel JM, Bestermann WH, Dyess EM, Graff A, Kursun A, Sutradhar S, et al. The Use of a Single-Pill Calcium Channel Blocker/Statin Combination in the Management of Hypertension and Dyslipidemia: A Randomized, Placebo-Controlled, Multicenter Study. The Journal of Clinical Hypertension. 2009;11(1):22–30.

7. Malekzadeh F, Marshall T, Pourshams A, Gharravi M, Aslani A, Nateghi A, et al. A pilot double-blind randomised placebo-controlled trial of the effects of fixed-dose combination therapy (‘polypill’) on cardiovascular risk factors. International Journal of Clinical Practice. 2010;64(9):1220–7.

8. Park JS, Shin JH, Hong TJ, Seo HS, Shim WJ, Baek SH, et al. Efficacy and safety of fixed-dose combination therapy with olmesartan medoxomil and rosuvastatin in Korean patients with mild to moderate hypertension and dyslipidemia: an 8-week, multicenter, randomized, double-blind, factorial-design study (OLSTA-D RCT: OLmesartan rosuvaSTAtin from Daewoong). DDDT. 2016 Aug 16;10:2599–609.

9. PILL Collaborative Group. An International Randomised Placebo-Controlled Trial of a Four-Component Combination Pill (“Polypill”) in People with Raised Cardiovascular Risk. PLoS One [Internet]. 2011 May 25 [cited 2019 Nov 20];6(5). Available from: https://www.ncbi.nlm.nih.gov/pmc/articles/PMC3102053/

10. Soliman EZ, Mendis S, Dissanayake WP, Somasundaram NP, Gunaratne PS, Jayasingne IK, et al. A Polypill for primary prevention of cardiovascular disease: A feasibility study of the World Health Organization. Trials. 2011 Jan 5;12(1):3.

11. The Indian Plycap Study (TIPS). Effects of a polypill (Polycap) on risk factors in middle-aged individuals without cardiovascular disease (TIPS): a phase II, double-blind, randomised trial. The Lancet. 2009 Apr 18;373(9672):1341–51.

12. Grimm R, Malik M, Yunis C, Sutradhar S, Kursun A. Simultaneous treatment to attain blood pressure and lipid goals and reduced CV risk burden using amlodipine/atorvastatin single-pill therapy in treated hypertensive participants in a randomized controlled trial. Vasc Health Risk Manag. 2010;6:261–71.

13. Zamorano J, Erdine S, Pavia A, Kim JH, Al-Khadra A, Westergaard M, et al. Proactive multiple cardiovascular risk factor management compared with usual care in patients with hypertension and additional risk factors: the CRUCIAL trial. Current Medical Research and Opinion. 2011 Apr 1;27(4):821–33.

14. Thom S, Poulter N, Field J, Patel A, Prabhakaran D, Stanton A, et al. Effects of a Fixed-Dose Combination Strategy on Adherence and Risk Factors in Patients With or at High Risk of CVD: The UMPIRE Randomized Clinical Trial. JAMA. 2013 Sep 4;310(9):918–29.

15. Selak V, Elley CR, Bullen C, Crengle S, Wadham A, Rafter N, et al. Effect of fixed dose combination treatment on adherence and risk factor control among patients at high risk of cardiovascular disease: randomised controlled trial in primary care. BMJ. 2014 May 27;348:g3318.

16. Patel A, Cass A, Peiris D, Usherwood T, Brown A, Jan S, et al. A pragmatic randomized trial of a polypill-based strategy to improve use of indicated preventive treatments in people at high cardiovascular disease risk. Eur J Prev Cardiolog. 2015 Jul 1;22(7):920–30.

17. Yusuf S, Pais P, Sigamani A, Xavier D, Afzal R, Gao P, et al. Comparison of Risk Factor Reduction and Tolerability of a Full-Dose Polypill (With Potassium) Versus Low-Dose Polypill (Polycap) in Individuals at High Risk of Cardiovascular Diseases. Circulation: Cardiovascular Quality and Outcomes. 2012 Jul 1;5(4):463–71.

18. Castellano JM, Sanz G, Peñalvo JL, Bansilal S, Fernández-Ortiz A, Alvarez L, et al. A polypill strategy to improve adherence: results from the FOCUS project. Journal of the American College of Cardiology. 2014;64(20):2071–82.
